# The Detection of Thread Roll’s Margin Based on Computer Vision

**DOI:** 10.3390/s21196331

**Published:** 2021-09-22

**Authors:** Zhiwei Shi, Weimin Shi, Junru Wang

**Affiliations:** School of Mechanical Engineering & Automation, Zhejiang Sci-Tech University, Hangzhou 310018, China; 201930506079@mails.zstu.edu.cn (Z.S.); swm@zstu.edu.cn (W.S.)

**Keywords:** detection of thread roll’s margin, computer vision, deep learning, Circle Gradient Operator, Keras, Kalman Filter

## Abstract

The automatic detection of the thread roll’s margin is one of the kernel problems in the textile field. As the traditional detection method based on the thread’s tension has the disadvantages of high cost and low reliability, this paper proposes a technology that installs a camera on a mobile robot and uses computer vision to detect the thread roll‘s margin. Before starting, we define a thread roll‘s margin as follows: The difference between the thread roll‘s radius and the bobbin’s radius. Firstly, we capture images of the thread roll‘s end surface. Secondly, we obtain the bobbin’s image coordinates by calculating the image’s convolutions with a Circle Gradient Operator. Thirdly, we fit the thread roll and bobbin’s contours into ellipses, and then delete false detections according to the bobbin’s image coordinates. Finally, we restore every sub-image of the thread roll by a perspective transformation method, and establish the conversion relationship between the actual size and pixel size. The difference value of the two concentric circles’ radii is the thread roll’s margin. However, there are false detections and these errors may be more than 19.4 mm when the margin is small. In order to improve the precision and delete false detections, we use deep learning to detect thread roll and bobbin’s radii and then can calculate the thread roll’s margin. After that, we fuse the two results. However, the deep learning method also has some false detections. As such, in order to eliminate the false detections completely, we estimate the thread roll‘s margin according to thread consumption speed. Lastly, we use a Kalman Filter to fuse the measured value and estimated value; the average error is less than 5.7 mm.

## 1. Introduction

We develop a low-cost and high-precision method to detect a thread roll’s margin. This method mainly uses a camera which is installed in a mobile robot to obtain images. Then, we calculate the margin of the thread roll by computer vision. This method can replace workers, which has great practical significance.

Currently, the textile industry in China is labor-intensive. We are facing the problems of low productivity and high labor costs [1]. According to the Ministry of Industry and Information Technology of the People’s Republic of China, the digitization rate of equipment in the textile industry is 36.06%. However, only 27.74% of all digital equipment is connected to the Internet. Thus, the overall level of automation is still low [2]. Therefore, with rising labor costs, the automation rate of China’s textile enterprises needs to be further improved, especially for small and medium-sized enterprises [1,2,3]. We propose a method which can help to improve productivity and safety, and also reduce dependence on skilled workers.

The main difficulties are as follows: The most important one is that the image background is very complex, so it is very difficult to extract the whole thread roll according to contours, colors, etc. In addition, the deep learning method may also have false detections; this is not allowed in factories. The second one is that the light may vary in a factory setting, so our method should eliminate the interference of light. Thirdly, the colors of thread vary, so our method should have broad adaptability. In addition to that, the conversion between image size and real size may lead to precision loss.

**Figure 1:**
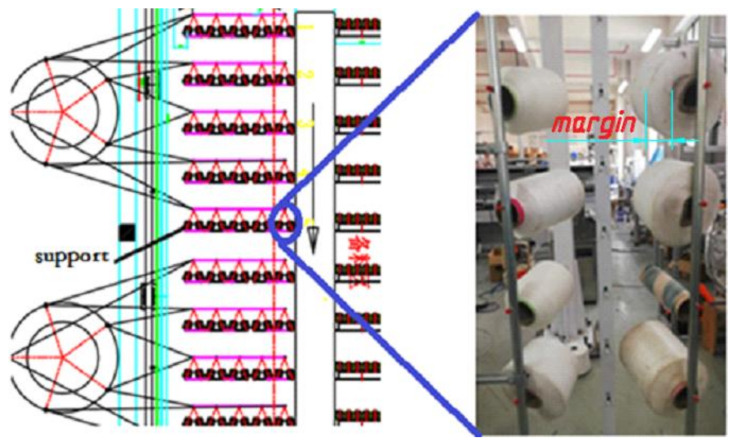
Supports array, there are 20 to 30 rows and every row has five supports. In every support, there are eight thread rolls distributed in two columns.

In these textile enterprises, the supports are usually distributed in 20 to 30 rows, and every row contains five supports. What we detect is the difference between the thread roll‘s radius and bobbin’s radius as shown in Figure 1 (red word “margin”). The processing steps can be described briefly below: The first step is putting the thread roll on supports by workers. Secondly, thread is put into the weaving machine, passing through the guide tubes. In the weaving process, workers patrol and see the thread rolls’ margins and replace the exhausted thread rolls. This method is not only inefficient but also produces other problems. For example, if the thread breaks because of the tension, this situation is difficult to be found in time, and can lead to serious problems. There are two types of methods that can help to monitor thread rolls: sensor–based methods and computer vision-based methods [3]. In previous studies, electronic sensors were mainly used to detect thread, but were mainly used to detect the thread’s defects, so these are not suitable to detect thread margins. In addition, there are thousands of thread rolls in one factory, so we need thousands of sensors, which is expensive. The other method is using computer vision. For example, Harbin University of Science and Technology developed a method to detect yarn based on an improved image threshold segmentation algorithm [4]. We find these methods are sensitive to the light. The most important factor is that the above methods cannot detect the margin of thread rolls.

In order to solve the above problems, we develop a method that only needs one mobile robot with a camera, which can monitor all thread rolls. The robot moves to the specified location, and then triggers the camera to capture an image. This method mainly uses image gradient and deep learning to obtain each thread roll’s margin as a measured value, then estimates the thread roll’s margin according to its thread consumption speed and fuses the measured value and estimated value by a Kalman Filter. This approach can completely eliminate false detections and the influence of light. This method is suitable for all kinds of thread rolls. In addition, this method can also reduce the precision loss that occurs when converting the margin in the image into a real value.

This work presents the following contributions: Firstly, we propose the first thread roll monitoring method in the world that can be used to detect a thread roll’s margin. Secondly, our method can completely eliminate false detection by combining deep learning and traditional computer vision methods. Thirdly, our method can be used for different kinds of thread rolls.

The rest of the paper is organized as follows: Section 2 is the related works. Section 3 presents the prepare works. Section 4 is the main part of this paper, it propose two ways to detect the thread roll’s margin. Section 5 gives the experiential results on different methods and Section 6 presents the conclusion and discussion of this study.

## 2. Related Works

According to our investigation, there is no one method or system that can be used to detect the margin of a thread roll. In previous studies, there are two kinds of methods used to detect whether the thread is exhausted: electronic sensors and CV technology. Electronic sensors are mainly used to detect thread, but not detect the thread roll’s margin; for example, some sensors detect the thread’s tension or vibration frequency, and can only be used to detect whether thread is used up. Computer vision is also used in textile industry [3,4,5,6,7,8,9,10,11]. However, it is mainly used to detect thread rolls’ defects [4,5,6,7,8,9,10,11]. It detects thread by extracting thread features, such as color, edge, etc. The main research is listed as follows.

### 2.1. Electronic Sensors

Imae M, Iwade T, and Shintani Y developed a thread-detection system based on tension [5]. They use pressure sensors to drive and regulate the speed of the let-off and take-up motor, to keep the yam tension dynamically stable within the allowed scope. At the same time, the system reads data from sensors. If there is no yarn, the pressure value is zero. Catarino A, Rocha A M, Monteiro J also developed a way to monitor knitting process through yarn input tension [6]. However, there are some problems, such as that the sensors add additional friction on the thread, so the thread may be broken by friction. Additionally, the signal is also unstable in some situations. In order to solve the above problems, Nanjing University of Aeronautics and Astronautics developed a non-contact thread detection system [7]; this system detects thread by using the relationship between the vibration frequency and tension of the moving thread. Firstly, they use high speed airflow to stir the thread. Then, they design a signal acquisition system to obtain the vibration frequency of the thread. Lastly, they use an equation to convert the frequency into the tension of the thread. This method can be used to detect whether the thread is exhausted, but not used to detect the margin. However, the implementation of this system is more complex, and the thread tension is very low in our conditions. As such, it may not detect the thread. In addition to that, Huang Suping, Zhao Lei, Zhang Shuaishuai, and YAO Guixiang proposed a method to detect the linear density of thread in a thread roll, but according to our tests, the accuracy is not high [8].

### 2.2. Computer Vision

There are some methods using computer vision, but it is mainly used to detect thread defects. For example, Harbin University of Science and Technology developed a method to detect yarn defects based on an improved image threshold segmentation algorithm [4]. They use improved bilateral filtering to obtain the yarn edge data and then use the optimal threshold calculation method to segment and locate the thread in an image. It can be used to detect thread, but is not suitable to detect the thread margin. This method requires that the background should be pure. Further, Li Jin-Fei, Li Jian-Qiang, Duan Yu-Tang, Ren Guo-Dong, and SHI Wei-Min propose a network of multi-scale depth separable convolution blocks modified based on the Inception-Resnet-A block of Inception v4 to detect yarn in pipes [9]. In addition to that, Zhao Yan, Zhang Huanhuan, Jing Junfeng, and Li Pengfei proposed a yarn defect detection method based on spatial fuzzy C-means (FCM) clustering. The above two methods are mainly used to detect thread defects [4] and help to detect whether the thread is exhausted.

The most important problem is that these methods cannot monitor the margin of thread. This can lead to serious results. If thread is exhausted, we have to stop the weaving machine and then tie the new thread and old thread. This is a complicated and difficult process. To solve these problems, this paper provides a low-cost and high-precision thread roll margin detected system; when the detected margin is less than a certain value we can prepare to replace the bobbin in advance.

## 3. Preparations

Before starting, there are two tasks that need to be done. The first one is to obtain the thread roll’s coordinates in an image. The second one is to establish the conversion relationship between the pixel size and actual size.

### 3.1. Getting Thread Roll’s Coordinates in Image

In order to detect the thread roll’s margin, the first step is to locate the bobbin in the image. We know that the bobbin in the image has a clear circular or elliptical edge [10,11,12]. We can locate the bobbin by detecting an ellipse or circle in the image. However, the current circle-detection algorithms, such as Hough-Circle Transform [11,12,13,14,15,16,17,18,19,20,21], have some false detection. As such, we use a new method to detect bobbins. The steps are as follows.

**Figure 2:**
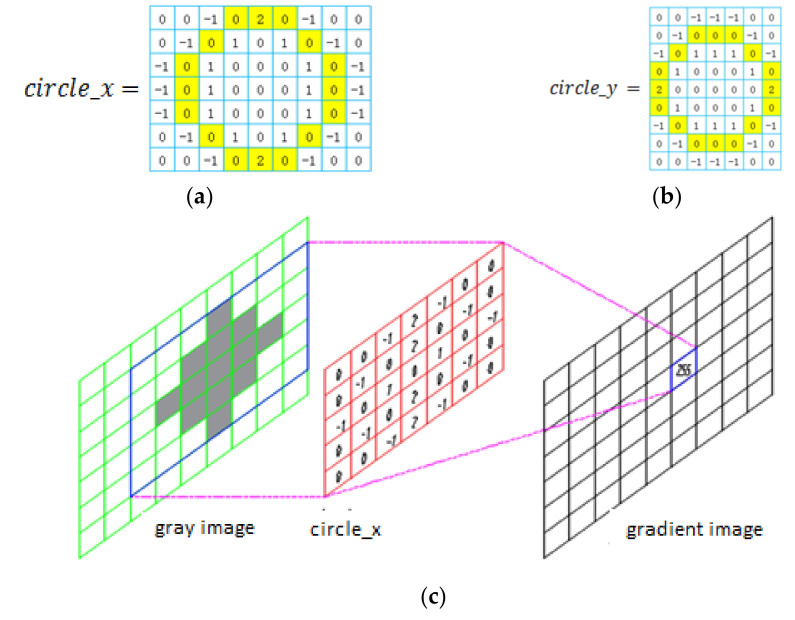
The convolution process use filters which are designed by the features of Roll. (**a**) circle_x filter. (**b**) circle_y filter. (**c**) shows the convolution process, where a gradient image is convolved with the grayscale image and circle_x or circle_y.

**Figure 3:**
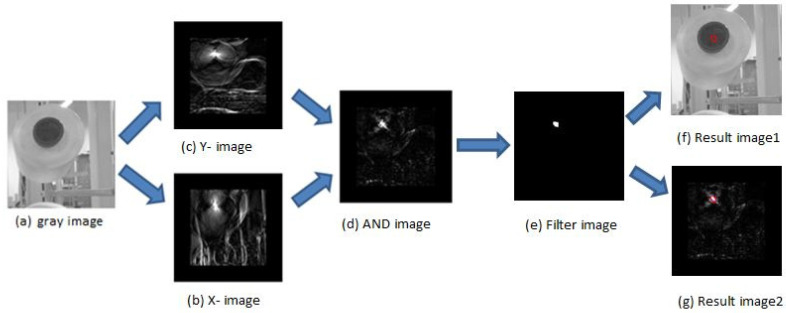
Convolution image, (**a**) is gray image. (**b**) is X-image. X-image is the grayscale image convoluted with circle_x. (**c**) is Y-image. Y-image is the grayscale image convoluted with circle_y. (**d**) is the AND image. The AND image is obtained by operating the AND operation between the X-image and Y-image. (**e**) is the filter image. The filter image is obtained by threshold and eroding and dilatinge the AND image. Lastly, we draw the detected center (red circle) of the bobbin in (**f**,**g**).

- Transform the raw image into a grayscale image;
- Design two circle-filters according to the bobbin’s features [13], circle_x and circle_y. The designing principles are that the convolution between the image and filter is zero when the image only has one color or has no change in gradient. For example, in Figure 2a,b are two circle-filters designed to detect a bobbin with a seven-pixel diameter in the image;
- The calculation of two-dimensional convolutions [13] in Figure 2c shows the calculating process with a five–pixel-diameter circle filter. It is known that the convolution sum is particularly large when the filters slide to the detected area;
- Obtain a convolution image [12]. Using sliding circle_x with fixed step in the grayscale image via computer convolution, we obtain a convolution image: X-img. Then, we obtain Y-img in the same way. The X-img and Y-img are shown in Figure 3;

- Use an AND operation to process the corresponding pixel value of the X-image and Y-image to obtain the AND image, as shown in Figure 3.
- We use the threshold function in opencv to process the AND image and then obtain a binary image. Next, we use a morphological filter to process the binary image to eliminate the noise, as shown in Figure 3e.
- We extract contours from the filter image, and the contour’s center is the center of the bobbin. The results are drawn in Figure 3f,g.

Finally, the bright area is the center of the bobbin, so we can locate all of the bobbin’s coordinates. In this process we design some circle-filters according to the bobbin’s shape in the image and then process the image by the above-mentioned method. The results show that the detection accuracy of this algorithm is more than 98.6%.

### 3.2. Perspective Transformation of Image

**Figure 4:**
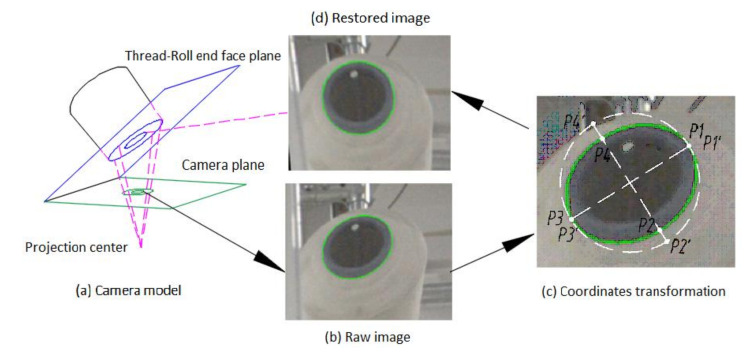
Shows the progress of perspective transformation. (**a**) The camera plane is not parallel to the bobbin’s end face plane, so it lead to the bobbin’s end face being ellipse in the raw image. (**b**) Bobbin’s end face is ellipse in raw image. (**c**) Calculating the vertex coordinates. (**d**) The transformed image, converting the ellipse into a circle.

The actual diameter of the bobbin is known, and its size in an image can be detected. As such, we can establish the conversion relationship between the real size and image size. Then, we can calculate the thread roll’s real diameter. However, the image captured by the camera is deformed, and the round bobbin will turn into an ellipse in the image because the camera plane is not parallel to the bobbin’s end face plane, as shown in Figure 4a. In order to establish the conversion relationship, the image needs to be restored using perspective transformation [14]. The conversion matrix is shown in Equation (1), where $\left(u,v\right)$ are the pixel coordinates in original image, and $\left(x=x^{\prime }/w^{\prime },y=y^{\prime }/w^{\prime }\right)$ are the corresponding pixel coordinates in restored image. From Equation (1), we know that if four original coordinates and its restored coordinates are obtained, we can obtain the transformation matrix and then restore the image. Figure 4b is the raw image and Figure 4d is the restored image. Transformation steps are as follows (we suppose that the bobbin edge has been detected and know the ellipse equation):

- Calculate the vertex coordinates $\left(P1,P2,P3,P4\right)$ of the ellipse in the raw image, which is shown in Figure 4c;
- The end face of bobbin is a circle, and its diameter in the transformed image is the major axis of the ellipse. As such, we can calculate the corresponding coordinates $\left(P1^{\prime },P2^{\prime },P3^{\prime },P4^{\prime }\right)$ of four vertexes in theory, which is shown in Figure 4c;
- Bring the above coordinates into Equation (1), producing Equation (2) [14], so we can obtain the conversion matrix and then produce a restored image, as shown in Figure 4d;
- Establish the conversion relationship between pixel size and real size via the bobbin’s pixel diameter and real diameter.
  (1) $$\left[\begin{array}{ccc} x^{\prime } & y^{\prime } & w^{\prime } \end{array}\right]=\left[\begin{array}{ccc} u & v & w \end{array}\right]\left[\begin{array}{ccc} a_{11} & a_{12} & a_{13}\\ a_{21} & a_{22} & a_{23}\\ a_{31} & a_{32} & a_{33} \end{array}\right],$$
  (2) $$\left[\begin{array}{ccc} 61*w^{\prime } & 39*w^{\prime } & w^{\prime }\\ 48*w^{\prime } & 100*w^{\prime } & w^{\prime }\\ 109*w^{\prime } & 113*w^{\prime } & w^{\prime }\\ 122*w^{\prime } & 52*w^{\prime } & w^{\prime } \end{array}\right]=\left[\begin{array}{ccc} 66 & 47 & w\\ 48 & 100 & w\\ 104 & 105 & w\\ 122 & 52 & w \end{array}\right]\left[\begin{array}{ccc} a_{11} & a_{12} & a_{13}\\ a_{21} & a_{22} & a_{23}\\ a_{31} & a_{32} & a_{33} \end{array}\right],$$

## 4. Proposed Methods

Before starting, we define the thread roll‘s margin as follows: The difference between the thread roll‘s radius and the bobbin’s radius. In this part, the authors propose two methods to detect the thread roll’s margin by computer vision. The first one is based on the thread roll‘s contours; the second one uses deep learning to calculate the thread roll’s margin. Then, we fuse the two results as a measured value. Finally, in this paper, we estimate the thread roll’s margin according to thread consumption speed and fuse the measured value and estimated value with a Kalman Filter.

### 4.1. Thread Roll’s Margin Detection by Contours

The above-mentioned method in Section 3.1 can detect the bobbin’s coordinates in an image. However, the bobbin’s edge is not detected accurately. Therefore, it is necessary to use a new method to detect contours.

This algorithm is to detect the thread roll’s margin by the following steps: Firstly, we convert the raw image into a gradient image. Secondly, we obtain a sub-image (200 × 200) of every thread roll. The center of each sub-image is the bobbin’s coordinates, obtained in Section 3.1. Thirdly, we detect the thread roll and bobbin’s contours. Fourthly, we fit these contours into an ellipse. Fifthly, we transform every sub-image of every thread roll by perspective transformation and then build the relationship between pixel size and actual size for every sub-image. Lastly, we obtain the actual size of the thread roll and calculate the thread roll’s margin.

#### 4.1.1. Image Process

**Figure 5:**
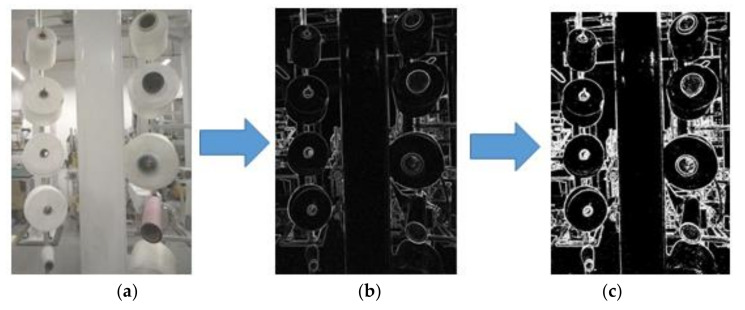
This figure shows image processing. (**a**) Raw grayscale image. (**b**) Gradient image. (**c**) Binary image.

In order to eliminate the influence of light and the colors of different thread rolls, we process the image by following steps: Firstly, we transform RGB image into a grayscale image. Then, we process the gray image with a Sobel [15,16,17,18,19,20,21] Filter and obtain a gradient image. Secondly, we convert the gradient image into a binary image [11,12,13,14,15,16,17,18,19,20,21], as shown in Figure 5. Lastly, we obtain sub-images (200 × 200) of every thread roll from the binary image. The center of the sub-image is the bobbin’s coordinates, obtained in Section 3.1.

#### 4.1.2. Contours Extracting and Processing

**Figure 6:**
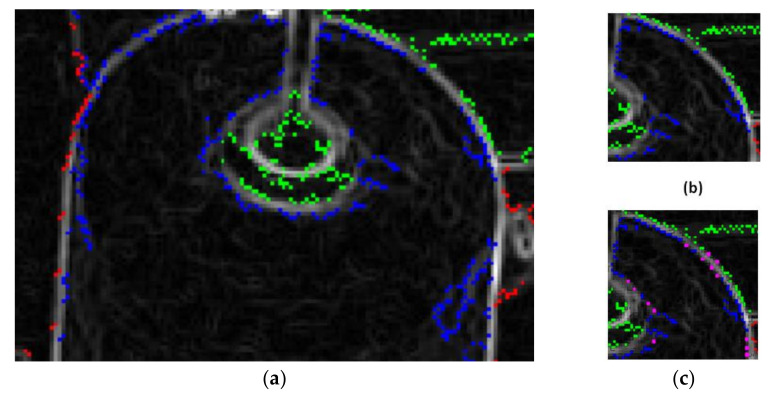
Points set. (**a**) Points in different set are marked in different color. (**b**) Points before interpolation. (**c**) Pink points are inserted points.

We extract contours from every sub-image by findContours [14] (a function in OpenCV). One detected contour is stored in different point sets. However, there are two problems here. The first one is that the contour points are dense in the zigzag place, but sparse in the smooth place, and the smooth place is just the edge of the bobbin and thread roll. Therefore, it is necessary to insert points at the sparse place. The other one is that one thread roll or bobbin’s edge points may not be in one points set, but distributed in two or more points sets. At the same time, all points in one set may detected from two or more different objects, as shown in Figure 6a. Therefore, before fitting an ellipse [18,19,20,21], it is necessary to segment and merge each points set.

To solve the first problem, we need to insert points in some places. The steps are as follows:

Calculate the average distance between adjacent points, as shown in Equation (3). n is the number of contour points;

(3) $$dis=\left(\overset{n-1}{\underset{i=0}{\sum }}\sqrt{{\left(x_{i+1}-x_{i}\right)}^{2}+{\left(y_{i+1}-y_{i}\right)}^{2}}\right)/n$$

- Calculate the distance between two adjacent points, as shown in Equation (4);
  (4) $$dis_{i}=\sqrt{{\left(x_{i+1}-x_{i}\right)}^{2}+{\left(y_{i+1}-y_{i}\right)}^{2}}$$
- If $10dis>dis_{i}>dis$, insert points between the i and i+1 point, then calculate the equation of the line between the two points; the line’s equation is Equation (5).
  (5) $$\left\{\begin{array}{ll} y=k_{1}x+b_{1} & \left|y_{i+1}-y_{i}\right|<\left|x_{i+1}-x_{i}\right|\\ x=k_{2}y+b_{2} & \left|y_{i+1}-y_{i}\right|>\left|x_{i+1}-x_{i}\right| \end{array}\right.$$
- Calculating the number of points to insert: $n=dis_{i}/dis$, then calculate the coordinates of the inserted points by Equation (6).
  (6) $$\left\{\begin{array}{c} x=x_{i}+t(x_{i+1}-x_{i})/n\;\;\;\;\;\;\;\;t=1,2,3\cdots ,n-1\\ y=k_{1}x+b_{1} \end{array}\right.$$

The result of inserting points is shown in Figure 6c.

To solve the second problem, we fit segment and merge point sets, and fit points into an ellipse. The algorithm of the fitting ellipse is as follows.

**Figure 7:**
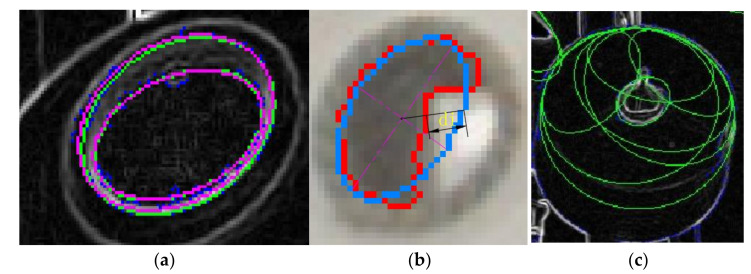
(**a**) Fit points into ellipse. (**b**) di is fitting error. (**c**) Fitting ellipse with some points in a set.

- The general equation of ellipse is as shown in Equation (7). Then, we obtain Equation (8) by putting the coordinates of points into Equation (7). Equation (7) can be written as Y = QM, $Q=\left[\begin{array}{c} \begin{array}{ccccc} A & B & C & D & E \end{array} \end{array}\right]$ Thus, we can obtain matrix Q from Equation (8). Then, calculate the parameters of the ellipse by Equation (9) [20], as shown in Figure 7a.
  (7) $$y^{2}=\left[\begin{array}{c} \begin{array}{ccccc} A & B & C & D & E \end{array} \end{array}\right]{\left[\begin{array}{c} \begin{array}{ccccc} x^{2} & xy & x & y & 1 \end{array} \end{array}\right]}^{T}$$
  (8) $$\left[\begin{array}{ccc} y_{1}^{2} & y_{2}^{2} & \cdots \end{array}\right]=\left[\begin{array}{c} \begin{array}{ccccc} A & B & C & D & E \end{array} \end{array}\right]\left[\begin{array}{ccc} x_{1}^{2} & x_{2}^{2} & \cdots \\ x_{1}y_{1} & x_{2}y_{2} & \cdots \\ x_{1} & x_{2} & \cdots \\ y_{1} & y_{2} & \cdots \\ 1 & 1 & \cdots \end{array}\right],$$
  (9) $$\left\{\begin{array}{l} X_{c}=\frac{-BD+2C}{4A+B^{2}}\\ Y_{c}=\frac{-BC-2AD}{4A+B^{2}} \end{array}\right.\;\;\;\;\;\;\;\left\{\begin{array}{l} a^{2}=\frac{2\left(AX_{c}^{2}-Y_{c}^{2}+BX_{c}Y_{c}-E\right)}{A-1+\sqrt{{\left(A+1\right)}^{2}+B^{2}}}\\ b^{2}=\frac{2\left(AX_{c}^{2}-Y_{c}^{2}+BX_{c}Y_{c}-E\right)}{A-1-\sqrt{{\left(A+1\right)}^{2}+B^{2}}} \end{array}\;\;\;\;\right.\theta =arctan\frac{B}{A+1},$$
- Error calculation. We calculate the average fitting error; that is, we calculate the average distance from the contour points to the edge of ellipse, as shown in Figure 7b. We suppose the coordinate of the contour point is $\left(x_{i},\;y_{i}\right)$. Then, we connect $\left(x_{i},\;y_{i}\right)$ and the center of the ellipse. The intersection point of the line and the ellipse is $\left(itsx,itsy\right)$, then $d_{i}$ is the absolute value of the distance between $\left(x_{i},\;y_{i}\right)$ and $\left(itsx,itsy\right)$, as shown in Equations (10) and (11). The average fitting error of ellipse is defined as Equation (12), where n is the number of points used for fitting;
  (10) $$\left\{\begin{array}{c} y=kx+b\\ y^{2}=Ax^{2}+Bxy+Cx+Dy+E \end{array}\right.\;\left\{\begin{array}{c} k=\left(y_{i}-Y_{c}\right)/\left(x_{i}-X_{c}\right)\\ b=y_{i}-kx_{i} \end{array}\right.,$$
  (11) $$d_{i}=min\left(\sqrt{{\left(x_{i}-itsx_{j}\right)}^{2}+{\left(y_{i}-itsy_{j}\right)}^{2}}\right)\;\;\;\;\;\;\;\;\;\;j=1,2,$$
  (12) $$error=\left(\overset{i=n}{\underset{i=0}{\sum }}|d_{i}|\right)/n,$$
- Lastly we calculate the coincidence degree. The coincidence degree is defined as Equation (13). It refers to the ratio between the number of fitted points and the circumference of the ellipse;
  (13) $$coid=n/\left(2\pi b+4\left(a-b\right)\right),$$

The next step is to segment and merge the points sets.

- Merge all points sets into one set;
- Using the first 10 points to fit ellipse, if the fitting error is small, add the next point to fit the ellipse until the fitting error is greater than a certain number. Then, we add these points into a new set, as shown in Figure 7c;
- Merge a different new set to fit the ellipse; if the fitting error is small, repeat the above step. We suppose these ellipses are a bobbin or thread roll;

The Table 1 shows some results of ellipse-fitting. We can draw the conclusion from the table and hundreds of tests that if the fitting error is less than 1.5 and the coincidence degree is greater than 0.3, these contours are considered as an elliptical boundary.

#### 4.1.3. Calculate Thread Roll’s Margin

The above-mentioned method can almost detect all bobbins and thread rolls. Then, for every detected bobbin, we transform the sub-image by the method mentioned in Section 3.1. As such, we can determine the actual diameter of every thread roll and then calculate their margins by Equation (14). $d_{1}$ is the thread roll’s diameter in the image, $d_{2}$ is the bobbin’s diameter in the image, and $D_{2}$ is the bobbin’s diameter in the real world.

(14) $$value1=\left(D_{2}*d_{1}/d_{2}-D_{2}\right)/2$$

### 4.2. Thread Roll’s Margin Detection by Deep Learning

There are some defects in the above method. Deep learning [22,23,24,25,26,27,28,29,30,31,32,33] can help to solve this problem. The general idea is: Training two models, model one is used to detect whether the thread roll’s margin is 0; otherwise, we enter model two to detect the thread roll’s margin. The models use Keras. Keras is a deep learning framework based on tensorflow [22,23,24,25]. After training the model, the margin can be obtained by inputting corresponding sub-image data. The network is composed of an input layer, hidden layer, activation function, and output layer. The activation layer is added after the hidden layer. The function of the activation layer is to add nonlinear factors. Otherwise, the linear transformation between input and output will be meaningless.

#### 4.2.1. Sample Selection and Processing

**Figure 8:**
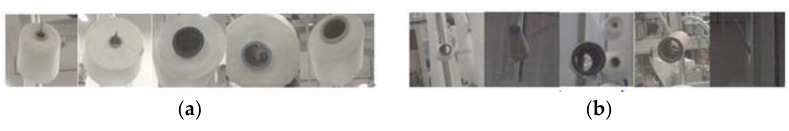
(**a**) Positive samples. (**b**) Negative samples.

Model one is a two-classification model; the samples include positive and negative samples [32,33], and the size is 200 × 200 × 1 pixels. The positive samples are sub-images in which the thread roll’s margin is not zero, as shown in Figure 8a, and it should cover all cases as much as possible. Negative samples are mainly images in which the thread roll’s margin is zero, and some background images, as shown in Figure 8b. Here, there are about 1000 positive samples, 1300 negative samples, and 200 valid samples. We use an image enhancement algorithm to extend the samples [32,33]. Then, the image is converted into a grayscale image, and finally normalized as the model one’s input. Typical positive and negative samples are shown in Figure 8.

#### 4.2.2. Construction of Network

**Figure 9:**
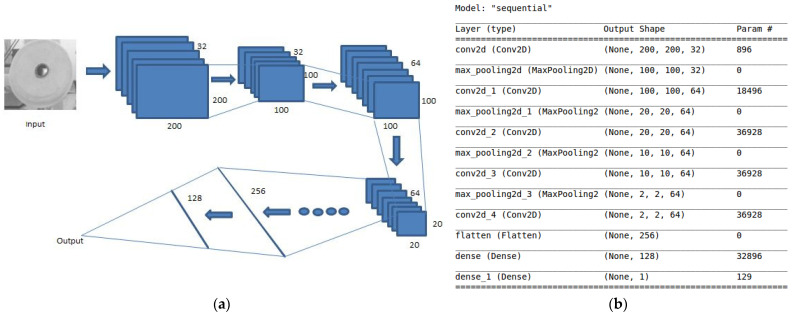
(**a**) Deep convolutional neural networks principles. (**b**) Model construction.

Model one [24,25,26,27,28,29] is used to distinguish whether the thread roll’s margin is 0, which is a classification problem. Therefore, a two-classification model is trained by multi-layer convolutional neural networks. The model construction is shown in Figure 9.

The first layer is convolution layer [25,26,27], it has 32 3 × 3 filters, and the activation function is relu [25,26,27], so the output size is 200 × 200 × 32. The convolution layer is mainly used to extract image features, such as gradient feature and so on. Here, the activation function relu is mainly used to add nonlinear factors; at the same time, it can overcome the gradient disappearance problem and speed up the training speed. The reason of adding nonlinear activation function is that if there is no activation function, each layer is equivalent to matrix multiplication. Linear transformation between input and output is meaningless. After adding the nonlinear activation function, the neural network can approximate almost any function. The equation of relu is $f\left(x\right)=max\left(0,x\right)$.

The second layer is a max pooling layer [25,26,27] that can reduce redundant information and highlight image features. After that, some convolution layers and max pool layers are added.

Next is the flatten layer [25,26,27], which makes the multidimensional data into one-dimensional data and realizes the transition from the convolution layer to the fully connected layer.

The last is the fully connected layer [25,26,27], which integrates features into a value to eliminate the influence of feature location on the results.

After the construction of model one, the following is done to select optimizer and loss functions. The task of machine learning is to optimize the parameters to minimize the result of the loss function. The loss function is the difference between the objective function value and the real value. The task of the optimizer is to calculate the gradient of the loss function in each epoch, and then update the parameters. Here, the optimizer selects Adam, which mainly calculates the first-order moment and the second-order moment, and obtains the new step size. For this two-classification problem, BinaryCrossentrop [27,28,29,30,31] is chosen as the loss function. There are only two categories (0,1). The expression of the loss function is Equation (15), where N is the number of samples and $y_{i}$ is the type of samples, negative sample is 0, positive sample is 1, and $p\left(y_{i}\right)$ is the probability that the i-th sample is a positive sample. This equation tells us that for each positive sample, it will add $logp\left(y_{i}\right)$ to the loss function, and for each negative sample, it will also add $log\left(1-p\left(y_{i}\right)\right)$ to the loss function. Therefore, the smaller the loss is, the more accurate the model is.

(15) $$J\left(w\right)=\frac{-1}{N}\overset{N}{\underset{i=1}{\sum }}\left[y_{i}*logp\left(y_{i}\right)+\left(1-y_{i}\right)*log\left(1-p\left(y_{i}\right)\right)\right]$$

#### 4.2.3. Model Training

**Figure 10:**
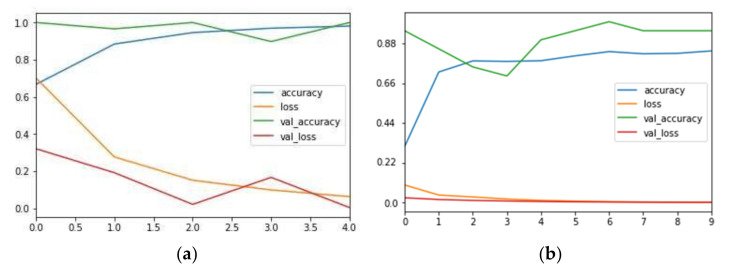
(**a**) Training result of model one. (**b**) Training result of model two. Transverse axis is the train steps, vertical axis is the accuracy (no more than 1).

Finally, after training the model one (the training results are shown in Figure 10a), the cumulative loss is 0.0637, the accuracy rate is 0.9807, the loss of validation samples is 0.0049, the accuracy rate of validation samples is 0.9985, and the model training results are good. After verification, the accuracy of model one’s output is 97.56%.

#### 4.2.4. Construction of Model Two

Model two is mainly used to detect each thread roll’s margin. Its input is same as model one. Its output is a six-dimensional array, which is the bobbin and thread roll’s radii and its coordinates in the image. The main training steps of model two are basically the same as model one. The main difference is that model two is a regression model, and the loss function is Equation (16) [27,28,29,30,31]. $X_{i,j}$ is the six-dimensional array marked by the sample, and $Y_{i,j}$ is the six-dimensional array output of the model prediction. Here, the smaller the loss is, the better the model training is. There are 1000 samples, and the final training result is shown in Figure 10b. The training accuracy is 0.8785, the cumulative loss is 0.017, the loss of validation samples is 0.0018, and the detection accuracy of validation samples is 0.9536. The results of model two training are good.

(16) $$f\left(i,j\right)=\overset{N}{\underset{i=1}{\sum }}\overset{6}{\underset{j=1}{\sum }}{\left(X_{i,j}-Y_{i,j}\right)}^{2}/N$$

After the models are trained, we use them to detect the margin. Firstly, we screen the sub-image (200 × 200) of every thread roll according to the coordinates obtained in Section 3.1. Secondly, we transfer the sub-image into a grayscale image and normalize it. Thirdly, we input it into model 1, which determines whether the thread roll’s margin is zero. If the margin is not zero, we continue to input the normalized image into model two; its output is the bobbin and thread roll’s coordinates and radii in the image. Finally, the actual thread roll’s margin is calculated by Equation (17), where $d_{1}$ is the thread roll’s diameter in pixels, $d_{2}$ is bobbin diameter in the image, and $D_{2}$ is bobbin diameter in the real world.

(17) $$value2=\left(D_{2}*d_{1}/d_{2}-D_{2}\right)/2$$

### 4.3. Fusing Detection Results

Each of the above two methods has its own advantages and disadvantages. The first method based on contours may have some false detection, and the result is not accurate when the thread roll’s margin is small. However, the second method based on deep learning may also has some false detection. If the site is changed, the samples may need to be updated and the model trained again. Therefore, in order to improve the detection accuracy, the two detection results need to be fused:

- If both results of the thread roll’s margin are zero, the thread roll’s margin is considered to be zero;
- If the results of both methods are not zero, the results need to be fused by Equation (18), where value1 is the thread roll’s margin detected based on detecting contours, value2 is the margin calculated by deep learning, and 150 is the maximum thread roll margin.
  (18) $$value3=value1*k+value2*\left(1-\left(value1+value2\right)/\left(150*2\right)\right)$$

### 4.4. Estimate the Thread Roll‘s Margin

It can be seen that the deep learning method and contour detection method both have false detections (the margin is not 0, but the detected value is 0). In order to prevent false detections, the following processing should be done: calculate the theoretical thread roll’s margin according to thread consumption speed and time interval. This result is the estimated value, the above-detected value based on computer vision is the measured value, and the two results are fused as the final result of thread roll’s margin through a Kalman Filter [34,35,36,37,38,39,40].

(19) $$x_{k}^{-}=\left[\begin{array}{c} value_{k}\\ v_{k} \end{array}\right]=\left[\begin{array}{cc} 1 & -t\\ 0 & 1 \end{array}\right]\left[\begin{array}{c} value_{k-1}\\ v_{k-1} \end{array}\right]+\left[\begin{array}{c} -0.5t^{2}\\ t \end{array}\right]u$$

(20) $$F=\left[\begin{array}{cc} 1 & -t\\ 0 & 1 \end{array}\right]\;\;B=\left[\begin{array}{c} -0.5t^{2}\\ t \end{array}\right],$$

(21) $$P_{k}^{-}=FP_{k-1}F^{T}+Q,$$

(22) $$K_{k}=P_{k}^{-}H^{T}{\left(HP_{k}^{-}H^{T}+R\right)}^{-1}$$

(23) $$x_{k}=x_{k}^{-}+K_{k}\left(Z_{k}-Hx_{k}^{-}\right)$$

(24) $$P_{k}=\left(I-K_{k}H\right)P_{k}^{-}$$

We suppose that the camera round time interval is T, the last result of the thread roll’s margin is $value_{k-1}$, and thread consumption speed is $v_{k}$. Five basic equation of Kalman Filter can be listed, as shown in Equations (19)–(24), where $Z_{k}$ is the thread roll’s margin and thread consumption speed measured by camera, $x_{k}^{-}$ is the estimated value of thread roll’s margin and thread consumption speed, and H = (1,0) is the observation matrix [34,35].

## 5. Experimental Results

We capture an image and convert it into a grayscale image and use two kind if methods to detect the thread roll’s margin; the results are as follows.

### 5.1. Thread Roll‘s Margin Detected by Method One

**Figure 11:**
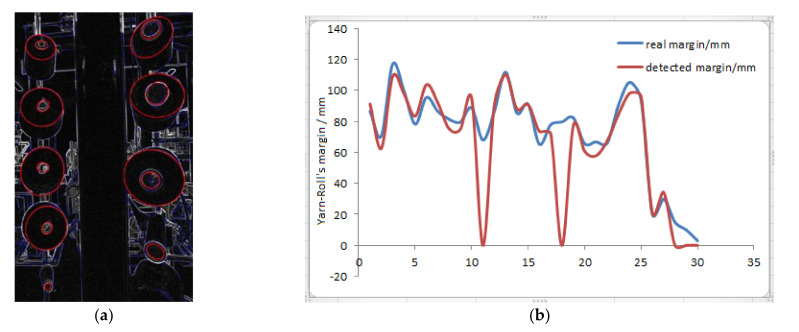
The detection results (**a**) The thread rolls and bobbins are detected and drawn in image. (**b**) The comparison between the real thread roll’s margin and the calculated margin. Red line is the margin detected from 30 different thread rolls, blue line is its real margin.

The thread rolls and bobbins are detected and drawn in Figure 11a, and then calculate the margin by Equation (14). The comparison between the real thread roll’s margin and the calculated margin is shown in Figure 11b. It can be seen that this method has obvious defects. When the thread roll’s margin is small, the detection error is large, and the maximum is more than 19.4 mm. When the thread roll’s margin is large, there are also false detections occasionally. Beside false detections, the average detection error is less than 7.2 mm, so it needs to be corrected.

### 5.2. Thread Roll‘s Margin Detected by Method Two

**Figure 12:**
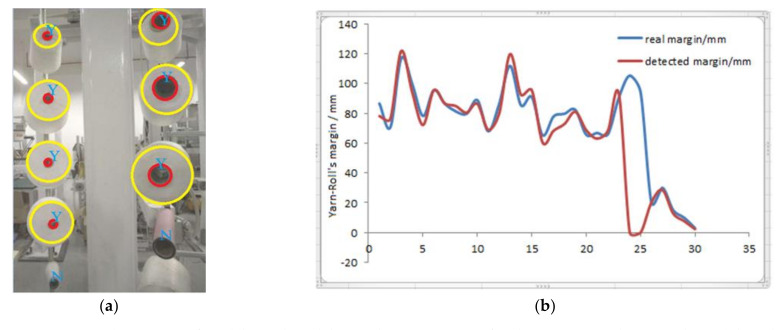
(**a**) The outputs of model 1 and model 2 are drawn in image. (**b**) The comparison between the real thread roll’s margin and the calculated margin. Red line is the margin detected from 30 different thread rolls, blue line is its real margin.

Firstly, we screen the sub-image (200 × 200) of every thread roll according to the coordinates obtained in Section 3.1. Secondly, we transfer each sub-image into a grayscale image and normalize it. Thirdly, we input it into model 1, which determines whether the thread roll’s margin is zero. If the margin is not zero, we continue to input the normalized image into model two; its output is the bobbin and thread roll’s coordinates and radii in the image. Finally, the actual outputs of the two models are shown in Figure 12a. N indicates that the thread roll’s margin is zero. Y indicates that the thread roll’s margin is not zero. The small red circle is the bobbin’s edge, and the big yellow circle indicates the edge of thread roll. Then, we calculate thread roll’s margin by Equation (17), according to the outputs of model 2. The result is shown in Figure 12b. According to the results, there are two false detections, and the others are accurate, with the error being less than 6.5 mm.

### 5.3. Fusing the above Two Results as Measured Value

**Figure 13:**
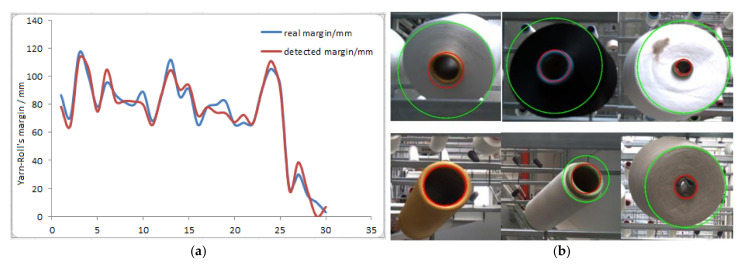
(**a**) The fused results. Blue line is the real margin and the red line is the detected line. (**b**) This is the measured values drawn in image.

The thread roll’s margin detection from an image is completed by the above steps. We fuse two values by Equation (18) to reduce false detection and improve the accuracy, and the average error is 6.7 mm as is shown in Figure 13a. However, we should realize that if false detection happens at the same time, the fused value is also a false detection. From Figure 13b, we can see that the above method can be used in different types of thread rolls, so this method has better generality.

### 5.4. Fusing Measured Value and Estimated Value by Kalman Filter

**Figure 14:**
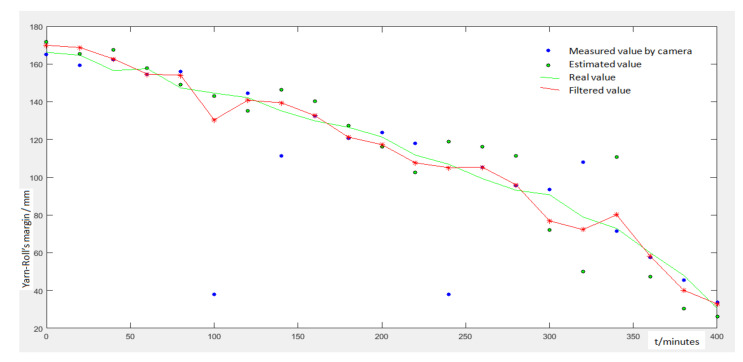
The final results detected in different time. Red line is filtered results, green line is real margin, green points are predicted value, blue points are measured value.

In order to verify the precision of results fused by Kalman Filter, we designed the following experiment: We put 30 thread rolls on supports, and the robot patrols and captures an image every 20 min. Then, we use the image to calculate each thread roll’s margin. Figure 14 shows the measured value and estimated value of one thread roll in different time. Then, we fuse the estimated value and measured value by Equations (19)–(24). We analyze all statistics and conclude the following results:

1. The false detections are eliminated completely;
2. Compared with the above value, the accuracy is improved a lot; the average error is less than 5.7 mm;
3. The thread consumption speed is not a constant value, so the estimated value may have a large deviation compared with the real value.

The above detection methods can initially meet the needs of enterprises and save a lot of costs.

## 6. Conclusions and Discussion

### 6.1. Conclusions

Currently, the main method of detection is using sensors to monitor whether the thread is used up. Compared with cameras, sensors have some disadvantages. For example, a camera can detect a thread roll’s margin. As such, it can predict when it will be used up, and the system can prepare to change the exhausted thread roll in advance.

In this paper, we present two kinds of methods that can detect thread roll’s margin. Method one is based on the gradient information of an image. Actually, thread rolls have many features, such as color, and we can also use color segmentation to extract the thread roll’s information. However, though actual tests, we find that the thread roll’s color in an image is severely influenced by light, but the gradient features of an image can eliminate the influence of light or background. Method two uses convolutional networks to detect the thread roll’s margin. Though actual tests, we find that the above two methods all have reasonable precision, and the processing time of every image is less than 80 ms. In order to eliminate false detection completely, we predict each thread roll’s margin as an estimated value according to its thread consumption speed. Then, we fuse the estimated value and measured value by Kalman Filter. By testing, the accuracy is improved a lot (the average error is less than 5.7 mm) and the false detections are eliminated completely.

### 6.2. Limination

The measuring range of Thread Roll’s is diameter is 10 cm to 40 cm. The range of camera’s distance to the thread roll is 50 cm to 150 cm. The range of the Lens Focal Length is from 3 mm to 8 mm. The average error is less than 5.7 mm when the distance from camera to thread roll is 65 cm, the Lens Focal Length is 6 mm.

This method can be used to solve other problems, for example, the neural networks can be used to detect other object. However, this method also has some limitations. For example, method 1 can only be used to detect a thread roll or some circular object. In addition to that, the samples of method 2 should cover all situations as much as possible. It is difficult. Thus, if we need to detect a totally different thread roll, we may have to add this thread roll’s samples and retrain the model.

### 6.3. Discussion

For future work, we have two directions of research. One direction is to reduce false detections.

**Figure 15:**
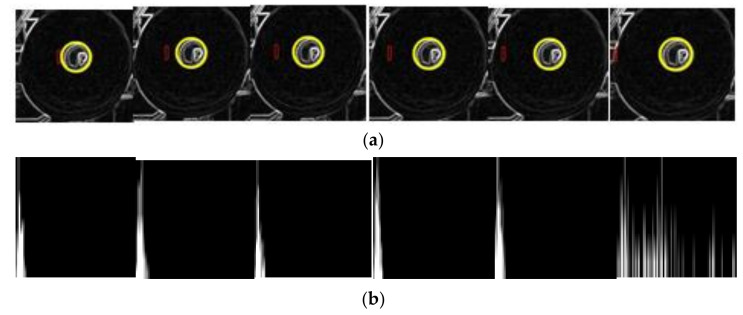
(**a**) The red rectangle is the area to calculate the gray value. (**b**) The gray-scale histogram of red rectangle.

For method one, we can use the gray values of the gradient image. The steps are as follows:

- Detect the edge of bobbin;
- Select a small rectangular area around the bobbin edge to calculate its gray value and grayscale histogram, and then move the small rectangle farther away from the bobbin and calculate its gray value and grayscale histogram, as shown in Figure 15a,b.
- We judge whether the red rectangle is in the edge of thread roll according to the gray value and grayscale histogram.

This can be used as an assisting method to improve accuracy, but is not yet tested. As such, we have to perfect this method.

For method two, we should optimize the model’s parameters and add samples to cover all cases as much as possible. Moreover, another further development consists of the integration of the other sensors that are used to detect the speed of thread consumption, so we can predict the margin with more accuracy. In this way, we will be able to test the performance of the proposed methods in real-time running scenarios.

## Figures and Tables

**Table 1:** This table shows the results of ellipse-fitting.

Points Number	Points Type	Ellipse Center	Minor and Major axis	Fitting Error	Coincidence Degree
117	Thread Roll	(120.6, 112.5)	44.7,55.5	1.03	0.36
121	Bobbin	(529.2, 48.3)	19.2, 30.1	0.99	0.74
96	Bobbin	(144.1, 778.2)	18.8, 23.7	1.15	0.69
214	Thread Roll	(141.3, 777.7)	75.5, 82.0	1.24	0.43
191	Thread Roll	(550.8, 285.3)	43.1, 47.6	1.23	0.66
37	False detection	(650.0, 818.5)	14.1, 27.3	2.72	0.26
59	Bobbin	(525.3, 855.4)	21.9, 26.3	1.35	0.38
140	False detection	(127.7, 562.6)	77.6, 82.2	0.98	0.27
